# Characterizing serial dependence as an attraction to prior response

**DOI:** 10.1167/jov.24.9.16

**Published:** 2024-09-26

**Authors:** Geoffrey K. Gallagher, Christopher P. Benton

**Affiliations:** 1 School of Psychological Science, University of Bristol, Bristol, UK

**Keywords:** serial dependence, orientation, visual perception

## Abstract

Serial dependence refers to a common misperception that can occur between subsequently observed stimuli. Observers misreport the current stimulus as being more similar to the previous stimulus than it objectively is. It has been proposed that this bias may reflect an attraction of the current percept to prior percept (Fischer & Whitney, 2014). Alternatively, serial dependence has also been proposed to be the result of an assimilative effect between observer decisions (Fritsche, Mostert, & de Lange, 2017; Pascucci, Mancuso, Santandrea, Libera, Plomp, & Chelazzi, 2019). Lying within this debate is the issue of how we *quantify* serial dependence. Should this be as a bias induced by prior stimuli or by prior responses? We investigated this by manipulating the orientation of the current stimuli such that they fell between previous stimulus and previous response. We observed an attraction to previous response and a concomitant repulsion from previous stimulus. This suggests that the attractive effect of serial dependence in orientation judgments is best quantified in relation to prior response.

## Introduction

Visual perception is an imperfect process that may be prone to systematic errors. These errors can sometimes tell us how vision works. When similar stimuli are viewed in sequence, observer reports of current stimulus features appear to be blended with the previous stimulus (Burr & Cicchini, 2014; Kiyonaga, Scimeca, Bliss, & Whitney, 2017). Fischer and Whitney (2014) showed this serial dependence effect occurring in judgments of orientation stimuli. When the orientation of Gabor stimuli observed on successive trials was within a narrow range, participants misreported the orientation of the most recent stimulus. The errors made by the participants suggested that their interpretation of the current stimulus was biased toward the stimulus they had observed previously.

A misperception might occur because the visual system uses the recent past as a rough guide to interpret the present. Our visual environment is generally not entirely static. Saccades or blinks constitute interruptions in visual input, and objects crossing the field of view can temporarily obstruct vision. Despite these short-term changes, elements of the environment may remain largely stable in the longer term, and we may be safe in assuming they have not changed (Dong & Atick, 1995; Girshick, Landy, & Simoncelli, 2011). If glare from a passing car windshield temporarily obstructs my vision, I can take for granted that the trees in front of me have not disappeared. The visual system may assume a degree of stability in our surroundings, extrapolating the current state of the environment based on previous information (Burr & Cicchini, 2014; Fischer & Whitney, 2014). Although this may be useful in smoothing over visual noise, it could also be the source of serially dependent errors.

To account for serial dependence in orientation judgements, Fischer and Whitney (2014) proposed that changes in neural gain or tuning resulting from a previous pattern of activation might affect the population code in response to the current stimulus, thereby biasing the resultant percept. By this account, changes in neural responsiveness are induced by sensory encoding, occurring as a result of stimulus exposure, prior to awareness or decisions. Prior stimulus values can therefore serve as a proxy for expected changes in responsiveness (Moon & Kwon, 2022). The idea that the residual neural trace from previous trials produces errors on the current trial is bolstered by evidence tying serial dependence to brain areas known to underlie visual processing. For example, results from functional magnetic resonance imaging (fMRI), which isolate serial dependence to early perceptual areas such as striate cortex (St. John-Saaltink, Kok, Lau, & de Lange, 2016), have been argued to support the view that serial dependence can occur at early stages of visual processing (Cicchini, Mikellidou, & Burr, 2017).

However, the occurrence of serial dependence across diverse stimulus types such as orientation (Fischer & Whitney, 2014; Pascucci, Mancuso, Santandrea, Libera, Plomp, & Chelazzi, 2019), facial identity (Liberman, Fischer, & Whitney, 2014), visual variance (Suárez-Pinilla, Seth, & Roseboom, 2018), body size estimation (Alexi et al., 2018), monetary value (Morimoto & Makioka, 2022), and aesthetics (Kim, Burr, & Alais, 2019) might instead support the idea of a general process affecting perceptual decisions rather than multiple low-level perceptual effects. Experimental results seem to support this idea. Ceylan, Herzog, and Pascucci (2021) demonstrated serial dependence across different depictions of orientation (Gabor and dot pattern stimuli) and suggested that this showed serial dependence operating at a higher level of processing, beyond basic stimulus features. Similarly, Morimoto and Makioka (2022) showed serial dependence not only between numerosity of coins but also between their value, a stimulus feature that would require processing at the level of abstract stimulus properties.

Other work has supported the idea of serial dependence occurring beyond brain regions sensitive to low level visual features. Sheehan and Serences (2022) observed attractive serial dependence in an orientation discrimination task; however, fMRI data obtained during this task indicated repulsive effects in early visual areas. The authors reconciled these findings by suggesting that serial dependence arises at higher level brain areas. Localizing serial dependence to brain regions that are not exclusively concerned with early sensory processing opens up the possibility that serial dependence might be non-perceptual. Indeed, multiple studies have argued that serial dependence may be a decision-based effect, not grounded in sensory processing (Ceylan et al., 2021; Fritsche, Mostert, & de Lange, 2017; Morimoto & Makioka, 2022; Pascucci et al., 2019).

In a decision-based account, rather than reflecting a perceptual blending between subsequently presented stimuli, serial dependence is instead the result of a post-perceptual attraction between decisions. Roughly put, successive stimuli do not actually look the same, but nevertheless we make similar decisions about them (although some decision-based accounts do allow for the possibility of perceptual change; see Pascucci et al., 2019).

The idea that serial dependence occurs at the level of decisions has been tested using trials where participants are not required to make a response, limiting the possibility of decisions in these trials causing serial dependence in the following trial. Fischer and Whitney (2014) found that lack of an explicit decision on one trial did not influence serial dependence in the following trial, a finding that was replicated in later studies (Czoschke, Fischer, Beitner, Kaiser, & Bledowski, 2019; Liberman et al., 2014; Manassi, Liberman, Chaney, & Whitney, 2017; Xia, Leib, & Whitney, 2016). These studies concluded that serial dependence is a passive perceptual effect that does not require a perceptual decision.

Of course, telling participants that they are not required to make a perceptual decision does not mean that they will not do so anyway, because of either the expectation of a response requirement or even just the repetitive nature of the task (Lau & Maus, 2019). In fact, experiments using slightly different methodologies, which might limit implicit decision-making, have found that serial dependence is reduced in the absence of an initial decision (Bae & Luck, 2020; Pascucci et al., 2019).

A major difficulty in investigating the distinction between stimulus- and response-based effects is that the two are often very highly correlated. This can make it difficult to disentangle the contributions of each to serial dependence. Fritsche et al. (2017) took an approach designed to measure participant perception in a way that dissociated decisions from stimuli. In a variation of an earlier experiment by Fischer and Whitney (2014), observers were asked to compare a presented stimulus to a simultaneously displayed standard and asked if they had the same orientation. One of these stimuli was vulnerable to serial dependence from a previous adjustment task. This approach meant that the participant's decision was no longer highly correlated with the specific value of the stimulus; repeating the same decision regardless of the underlying stimulus would not produce an assimilative effect resembling the proposed perceptual blending. The authors found that previous stimuli actually exerted a repulsive effect and that any assimilation observed was likely due to carryover of previous decisions. Other work has expanded upon this idea and produced models of how decision-based serial dependence might occur (Bosch, Fritsche, Ehinger, & de Lange, 2020; Pascucci et al., 2019).

Despite the evidence for this view, there remain some experimental results suggesting that serial dependence might represent an attraction to previous stimuli rather than being a decision-based effect. When Cicchini et al. (2017) replicated the experiment of Fritsche et al. (2017) with a wider range of differences between subsequent stimuli, they found that assimilative effects were evident in the equivalence task. This contrast in results was explained by the ideal context for serial dependence: Large stimulus differences should favor repulsive effects to aid discrimination, whereas small stimulus differences are less likely to represent meaningful change and so might be expected to be smoothed over by assimilative effects (Fischer & Whitney, 2014).

Cicchini et al. (2017) carried out another task to further test the basis of serial dependence. Participants were asked to reproduce observed stimulus orientations; however, on even-numbered trials, participants had to produce “mirror-flipped” responses, where the angle to be reproduced was flipped around the vertical axis (e.g., the correct response to a 45° stimulus would be 315°). This could be taken to suggest that prior decision is not the driver of serial dependence, as the response given was substantially different but serial dependence still occurred. However, it is not difficult to imagine a sequence of decisions leading to this result: Produce your decision on stimulus orientation (affected by any decision bias), then mirror it (Pascucci et al., 2019). The paradigm of Cicchini et al. (2017) does rule out any aspect of the physical response, such as motor biases, but cannot rule out decision effects.

Statistical modelling approaches have also been used to try to test the relative contribution of prior stimulus and response to serial dependence. Several authors (Fründ, Wichmann, & Macke, 2014; Moon & Kwon, 2022; Pascucci et al., 2019; Sadil, Cowell, & Huber, 2024; Sheehan & Serences, 2023; Zhou et al., 2024) have included previous stimulus and response as separate factors in models, demonstrating that response appears to be the better predictor of serial effects.

Previously, we discussed a number of studies that showed that serial dependence can occur in the absence of previous response. The findings of these no-response studies seem to fit well with a stimulus-driven account of serial dependence. However, if we can accept that implicit decisions may be made in no-response trials (Kim & Alais, 2021; Pascucci et al., 2019), then it remains possible that it is decision that drives serial dependence, and that the response (where one is made) is a better metric of that decision than the original stimulus.

In the current study, we used a novel method to attempt to demonstrate experimentally that an attractive effect from the previous trial may best be quantified as attraction to the previous response rather than the previous stimulus. We did this by creating a subset of trials where the current stimulus was placed midway between the previous stimulus and previous response. By pitting the two in opposition, we unambiguously demonstrated that, in our orientation task, serial dependence should be quantified as an attraction to prior response rather than prior stimulus.

## Materials and methods

All experiments were approved by the Psychological Science School Research Ethics Committee at the University of Bristol.

### Participants

Seventeen participants took part in this experiment (age range, 19–37 years; mean ± *SD* = 26.05 ± 4.92 years; nine females, eight males). Following a previous procedure (Gallagher & Benton, 2022), one participant was excluded from analysis due to poor performance (correlation between stimulus and response of less than 0.5). All participants provided informed consent and were free to withdraw from testing at any time. Participants were entered into a prize drawing for taking part in this study. All participants had normal or corrected-to-normal vision.

### Stimuli

The stimuli used in the present study were identical to those used by us previously (Gallagher & Benton, 2022). Briefly, stimuli were generated by applying an orientation filter to 1/*f* noise (Field, 1987). Our orientation filter was applied in the Fourier domain and consisted of two von Mises distributions mirrored around 180°. The concentration of the von Mises distributions was set to 4 to reproduce the low noise stimuli used in our previous study. Stimuli were contained in a 1.5° *SD* Gaussian envelope. Stimulus orientation was chosen randomly (using the MATLAB rand function; MathWorks, Natick, MA) from 0° to 179° in steps of 1°.

To reproduce the orientation of stimuli, participants were required to rotate the position of two white dots (width ∼1° of visual angle) so that if an imaginary line were drawn between these dots it would match the orientation of the stimulus. These dots occupied opposing poles of the area previously occupied by the orientation stimulus. Adjustment dots were initially displayed at a random orientation (0°–179° in steps of 1°). A 1° fixation circle at the center of the screen gave participants a measure of their progress through the trials. This circle was gray with a white outline. The inner gray area of the fixation circle was gradually filled in with black from its top pole as trials progressed.

Stimuli were presented on a 24-inch VIEWPixx/3D Lite monitor (VPixx Technologies, Saint-Bruno-de-Montarville, QC, Canada) with a resolution of 1920 × 1080 and a refresh rate of 120 Hz. All experimental scripts were created in MATLAB 2019b using the Psychophysics Toolbox for MATLAB (Brainard, 1997; Kleiner et al., 2007; Pelli, 1997). Stimuli were viewed from approximately 57 cm in a darkened room.

### Procedure

**Figure 1.**
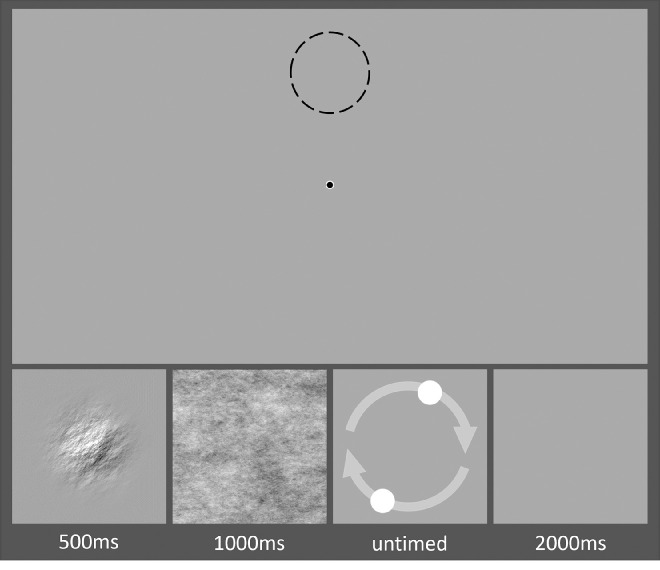
Typical trial sequence for the adjustment response portion of the noise experiment. The top image shows the screen as seen by a participant; the stimuli (shown below) appear in the dashed circle, and the fixation circle was present in all screens. The orientation stimulus appeared on-screen for 500 ms. Full screen noise was then displayed for 1000 ms to remove any visual aftereffects. The adjustment stimulus was then displayed in the same position previously occupied by the orientation stimulus. A blank screen was then displayed for 2000 ms. The next trial sequence appeared at a point 45° counterclockwise of the previous trial sequence (dashed circle).

Participants completed 500 trials of an adjustment task with breaks permitted every 100 trials. On each trial, participants saw a randomly oriented stimulus, the orientation of which they were then required to replicate. To begin the trial, an orientation stimulus was displayed for 500 ms. A full-screen 1/*f* noise mask was then displayed for 1000 ms to eliminate visual aftereffects. Following this, the response stimulus appeared at the same on-screen coordinates as the experimental stimulus. Immediately after the participant's response, a gray screen was displayed for 2000 ms before the next trial began (trial sequence shown in Figure 1). Participants took roughly an hour to finish this task.

**Figure 2.**
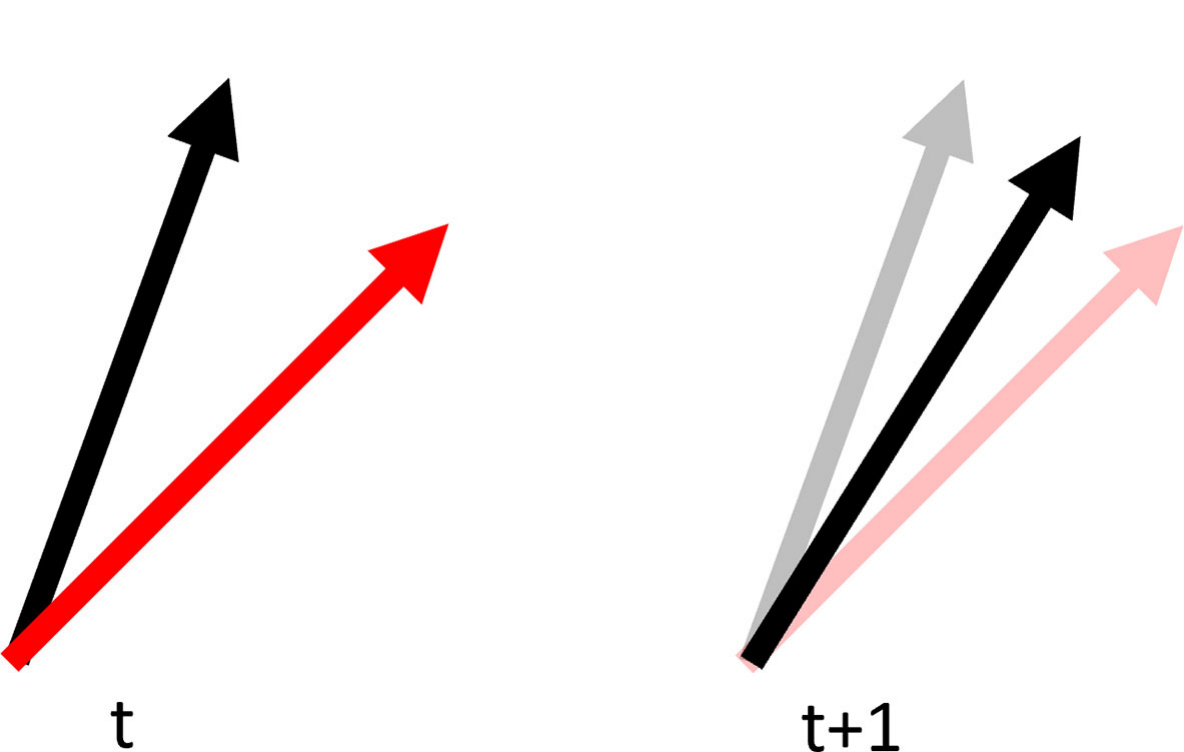
Diagrammatic representation of oppositional trial generation. When an error made in response orientation (red arrow) to a stimulus orientation (black arrow) on trial *t* was of sufficient magnitude (between 10° and 30°), the stimulus orientation (black arrow) on trial *t* + 1 was intermediate between the prior stimulus orientation and prior response orientation.

To distinguish the effect of previous stimulus and previous response, a subset of trials displayed stimuli with an orientation halfway between the previous stimulus and previous response (see schematic representation in Figure 2). We refer to this subset as “oppositional trials.” If participant errors on a trial were between 10° and 30°, the stimulus on the following trial was placed halfway between the prior stimulus and response. This range of errors was chosen so that differences would be large enough to produce serial dependence of a reasonable magnitude, but not so large as to reflect errors made due to attentional lapses. Oppositional trials were permitted to follow prior oppositional trials; however, this was limited by the conditions placed on the occurrence of these trials (within 10° and 30° error).

Orientation and response stimuli were displayed at points on an invisible circle of radius 9° of visual angle, which enclosed a central fixation circle (see top panel of Figure 1). Participants were instructed to maintain fixation throughout the experiment. Each orientation and response stimulus pair was presented 45° counterclockwise from the previous trial pair. Moving the stimuli between spatial positions was intended to minimize retinotopic adaptation (Knapen, Rolfs, Wexler, & Cavanagh, 2010). Maintaining a predictable path of stimuli was intended to promote serial dependence (Liberman, Zhang, & Whitney, 2016).

Responses were made using a Microsoft SideWinder controller (Microsoft Corporation, Redmond, WA). The shoulder buttons were used for rotation of the adjustment dots. Participants could switch between two different rotation speeds using a button on the face of the controller. Fast rotation allowed participants to rapidly align the response dots near the perceived orientation. Slow rotation allowed them to calibrate their response more precisely. When the dots had been moved into position, a separate button was pressed to confirm and progress to the next trial. Practice trials before the main experiment allowed participants to get used to the controls and the procedure of the experiment. Data from these practice trials were discarded.

### Analysis

**Figure 3.**
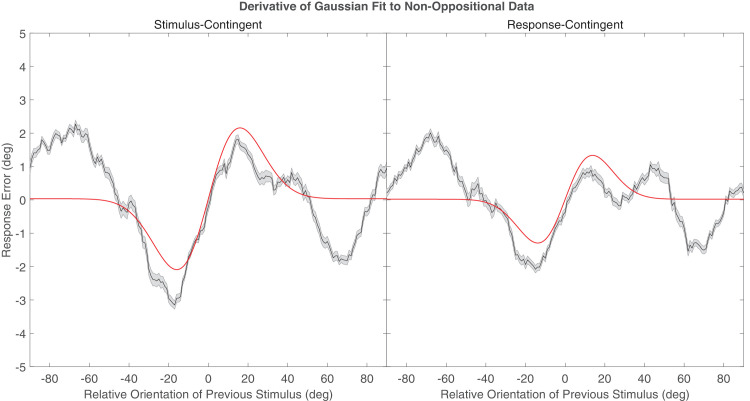
The commonly used DoG fit to non-oppositional data produced in this study. The left graph shows stimulus-contingent data, and the right graph shows response-contingent data. Note this is only intended as a visual comparison to previous studies. This approach was not appropriate for further analysis.

Oppositional and non-oppositional trials were analyzed separately. We first used the non-oppositional trials to demonstrate the standard attractive effect of serial dependence; we then used the oppositional trials to determine whether prior stimulus or prior response best characterized the attraction to the previous trial. Data from this experiment were analyzed using a model-free method (described below). This approach was favored over typical modeling approaches, such as the derivative of Gaussian (DoG) model (Fischer & Whitney, 2014), as the limited range of orientation differences in the oppositional trials would be insufficient to constrain a standard DoG curve fit (see Figure 3 for a DoG fit to non-oppositional data produced in this experiment; for the curve-fitting method, see Gallagher & Benton, 2022).

In the oppositional trials, previous stimulus and response were equidistant in orientation from the current stimulus. This equal spacing from the current orientation meant that the bias from each feature of the prior trial would mirror the bias from the other. The important point to take away from this bias analysis is which feature (previous stimulus or response) showed a bias of the same valence as any bias observed in the non-oppositional trials. As noted earlier, oppositional trials occurred after trials where participant error was between 10° and 30°. This means that, for oppositional trials, the orientation difference from the previous stimulus/response was constrained to the range of 5° to 15°. To ensure a fair comparison, the same range of data from non-oppositional trials was selected for analysis.

Preprocessing of data was carried out according to previously established procedures (Fritsche et al., 2017; Gallagher & Benton, 2022; Pascucci et al., 2019). Rotational response biases were removed by subtracting the circular mean of response errors from raw response errors for each participant (Fritsche et al., 2017). Large errors (>3 *SD* from the mean) were removed. Impossibly fast response times (<200 ms) and large response times (>3 *SD* from the mean) were also removed. The average percentage of trials removed by these procedures was 8.65%.

In order to investigate any biases arising from the prior stimulus and prior response, data were analyzed using both stimulus- and response-contingent approaches (Fritsche, 2016; Gallagher & Benton, 2022). Stimulus-contingent analysis calculates the difference between successive trials based on current and previous stimuli. Response-contingent analysis instead uses the previous response as a measure of stimulus orientation on the previous trial (Fritsche, 2016).

Response-contingent analysis may produce the appearance of serial dependence due to a general oblique response bias (Fritsche, 2016). In order to correct for this bias, a technique used by Pascucci et al. (2019) was applied to response-contingent data as outlined in previous work (Gallagher & Benton, 2022). A sum of sine waves model was fit to the plot of participant errors against orientation (fitting was performed using MATLAB fit function with “normalize” set to “on” as per the methods of Pascucci et al., 2019). Subtracting the predictions of this model effectively removes the influence of the oblique response bias. This procedure is hereafter referred to as “residualization.” This correction is not typically applied to stimulus-contingent analysis, as the distal stimulus is clearly not affected by response bias.

It is possible that an oblique response bias might have affected stimulus-contingent analysis, due to the way stimulus orientation was generated in this experiment. In oppositional trials, the difference between the previous stimulus and the current stimulus is partially determined by the previous response; the current stimulus is halfway between the prior stimulus and response. Any bias toward prior response is necessarily opposed to prior stimulus. Because prior response and current response are likely to be oblique, any attraction to stimulus might be reduced by the oblique response bias. A control analysis (described below) was performed to detect the presence of any residual biases not related to serial dependence. Stimulus-contingent corrected data are shown in the Supplementary Material.

### Model-free analysis

This model-free approach involves calculating the median error over the range of stimulus differences under which serial dependence is believed to occur. The median error over the range 0° to –45° is subtracted from the median error over the range 0° to 45° to produce an overall bias value for each participant. A positive bias value indicates assimilation, and a negative value repulsion (Samaha, Switzky, & Postle, 2019). Model-free biases were calculated for oppositional trials, where the stimulus was placed between the prior stimulus and response, as well as for the remaining non-oppositional data. Hedges’ *g* was calculated as a measure of effect size (Ellis, 2010).

Permutation tests were used to assess the statistical significance of model-free bias values. In a procedure that was repeated 10,000 times, the sign of a random subset of each participants data was reversed before model-free biases were calculated. We then calculated *p* values as the proportion of permutations producing bias values of greater magnitude than values calculated from the original data. The confidence intervals shown in our graphs were generated by bootstrapping (Efron & Tibshirani, 1994). For each of the 10,000 iterations, a random selection (with replacement) of participants was used to create a new sample from which bias values were recalculated.

## Results

### Oppositional trial generation

The experimental method described above generated a mean number of 144 oppositional trials per participant (*SD* = 36). As noted, it was possible for oppositional trials to occur in succession. The mean percentage of oppositional trials which followed a prior oppositional trial was 27% (*SD* = 8.4%).

### Serial dependence analysis

**Figure 4.**
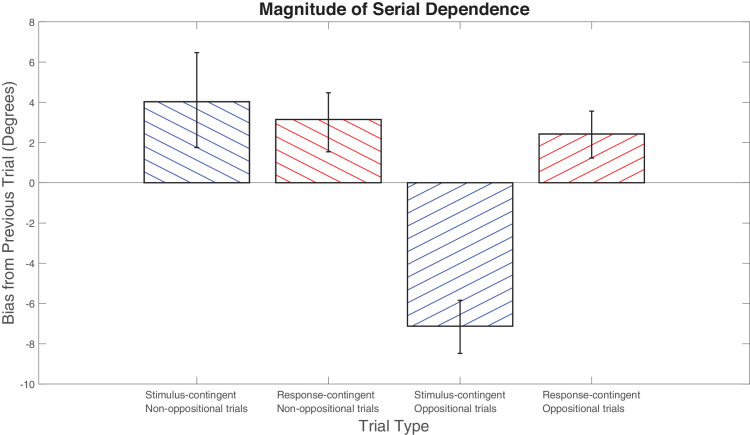
Response- and stimulus-contingent biases for oppositional and non-oppositional trials. Error bars represent 95% confidence intervals. Response-contingent analysis was corrected by residualization.

In non-oppositional trials, we observed a positive serial bias for both stimulus- and response-contingent analyses (stimulus-contingent bias = 4.03°, *p* = 0.0054, *g* = 0.77; response-contingent bias = 3.14°, *p* = 0.0012, *g* = 0.94) (see Figure 4). This demonstrates the standard attractive effect seen in orientation serial dependence. We can look to oppositional trials to see which of the two factors, previous stimulus or response, causes this assimilative bias. Response-contingent oppositional data showed a positive bias (bias = 2.42°, *p* = 0.0012, *g* = 0.94), whereas stimulus-contingent oppositional trial data showed a negative bias (bias = –7.13°, *p* < 0.001, *g* = 2.49). These results suggest that the positive effect observed in non-oppositional trials is best characterized as an attraction toward the prior response.

### Checking the oblique bias correction

A general bias toward making oblique responses may produce spurious serial dependence in response-contingent analysis (Fritsche, 2016; Pascucci et al., 2019). To eliminate this potential confound, we applied a correction to the data as described above. To check that this correction was successful, we ran an alternate flip trial analysis (Gallagher & Benton, 2022). This involves inverting the order of even-numbered trials to remove any temporal order effects. If an experiment consisted of 100 trials, trial 2 would be swapped with trial 100, trial 4 with trial 98, and so on. The oblique response bias can produce spurious serial dependence regardless of any real relationship between trials. We would therefore expect to still observe “serial dependence” in uncorrected flip trial data but not when the correction is applied.

**Figure 5.**
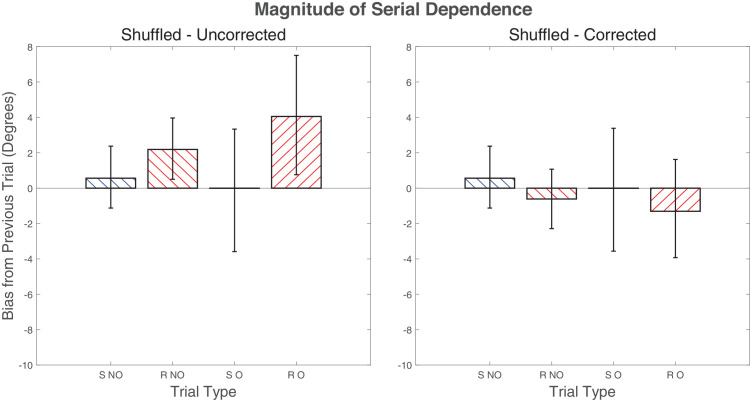
Biases in shuffled control data. The left graph shows data without oblique bias correction, and the right graph shows the same data with correction applied. S NO, stimulus-contingent, non-oppositional trials; R NO, response-contingent, non-oppositional trials; S O, stimulus-contingent, oppositional trials; R O, response-contingent, oppositional trials.

Figure 5 shows the results of this analysis. Spurious serial dependence is evident in uncorrected response-contingent alternate flip data (non-oppositional stimulus-contingent bias = 0.56°, *p* = 0.99, *g* = 0.14; non-oppositional response-contingent bias = 2.18°, *p* = 0.03, *g* = 0.56; oppositional stimulus-contingent bias = 0.00°, *p* = 0.55, *g* < 0.01; oppositional response-contingent bias = 4.05°, *p* = 0.04, *g* = 0.53). Applying residualization removes this effect (non-oppositional stimulus-contingent bias = 0.56°, *p* = 0.55, *g* = 0.14; non-oppositional response-contingent bias = –0.61°, *p* = 0.50, *g* = 0.16; oppositional stimulus-contingent bias = 0.00°, *p* = 0.99, *g* < 0.01; oppositional response-contingent bias = –1.30°, *p* = 0.39, *g* = 0.21). As expected, these results confirm that residualization is necessary only for response-contingent analysis.

### Effect of two-back stimulus

An apparent attraction toward the response from the previous trial could be determined by a strong two-back serial effect. For example, it could be the case that an apparent attraction to the one-back response, clockwise of the current stimulus, is in fact an attraction to the two-back stimulus also lying clockwise of the current stimulus. Consider the following sequence of trials:

$$\begin{array}{c} \;\begin{array}{c} n-2:\text{stimulus}=45^{\circ },\text{response}=45^{\circ }\\ \text{Pre-oppositional}\;\text{trial}\;n-1:\text{stimulus}=0^{\circ },\\ \;\text{response}=30^{\circ }\\ \text{Oppositional}\;\text{trial}\;n:\text{stimulus}=15^{\circ },\text{response}=20^{\circ } \end{array} \end{array}$$

If the stimulus and/or response on the *n* – 2 trial produce a large attraction (causing the 10°–30° difference between stimulus and response on the *n* – 1 trial necessary to produce an oppositional trial), it might also be responsible for the attractive effect observed in the following oppositional trial.

**Figure 6.**
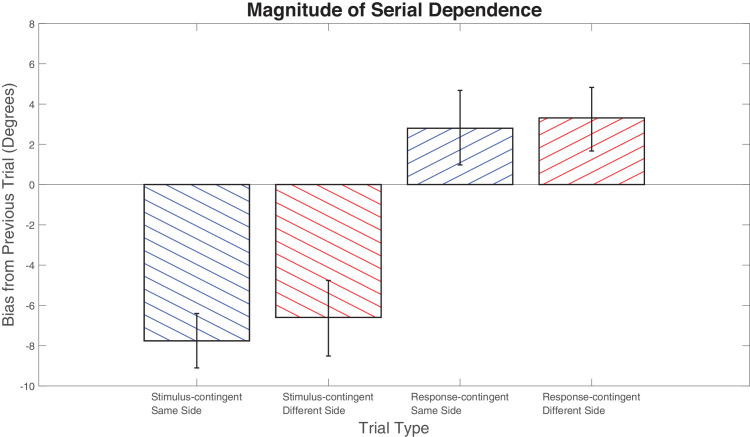
Stimulus- and response-contingent oppositional trials, sorted on the basis of whether the two-back stimulus is on the same side of the current stimulus as the one-back stimulus or not. Response bars represent 95% confidence intervals.

This presents a potential problem for interpretation of the current results. If we observe an attraction to the prior response, then we must check that this has not been caused by an attractive effect of the two-back stimulus. To assess this possibility, we split the results for stimulus- and response-contingent analysis based on whether the two-back stimulus was on the same side as the one-back stimulus or on the opposing side. If we observe a different pattern of results for data with the stimulus on the opposing side this suggests that the observed effect was driven by attraction to the two-back stimulus. The results demonstrate that the sign of the serial bias is not affected by whether the two-back stimulus was on the same side as the one-back stimulus: Response-contingent analysis still produces a positive effect regardless of whether the two-back stimulus was on the same side of the current stimulus or not (stimulus-contingent two-back same-side bias = –7.76°, *p* < 0.001, *g* = 2.52; response-contingent two-back same-side bias = 2.80°, *p* = 0.01, *g* = 0.67; stimulus-contingent two-back different-side bias = –6.60°, *p* < 0.001, *g* = 1.57; response-contingent two-back different-side bias = 3.31°, *p* < 0.001, *g* = 0.94) (Figure 6). It appears that two-back effects of stimulus are not responsible for the results observed in the main analysis.

### Response attraction resembles stimulus repulsion

Because of the opposition of stimuli on the test trials, it is possible that responses that appear to be the result of attraction toward the prior response could also have been due to repulsion from the prior stimulus. Both repulsion from the stimulus and attraction toward the response could produce a bias value with the same value due to the oppositional nature of the previous stimulus and response in test trials.

However, when considering non-oppositional trials (all other trials), we observed positive serial dependence in both response- and stimulus-contingent analyses. Stimulus and response will naturally be correlated in non-oppositional trials (mean correlation between stimulus and response for non-oppositional trials = 0.79, *SD* = 0.14). These trials demonstrate the effect of serial dependence in normal circumstances, without our oppositional manipulation. In this case, we observe positive serial dependence. This attractive effect in trials where stimulus and response are highly correlated suggests that response attraction in oppositional trials is not driven by stimulus repulsion.

The negative bias apparent in stimulus-contingent analysis of oppositional trials does not imply repulsion from previous stimuli because stimulus and response were set up in direct opposition. Stimulus- and response-contingent analysis in oppositional trials is only informative about which element of the prior trial is responsible for whatever bias we see in non-oppositional trials. The other element will always be directly opposed. If the effect observed in non-oppositional trials had been repulsive, then we would have concluded that the stimuli elicited a negative serial effect with no implication of attraction from the response.

It is worth noting that these biases will not perfectly mirror each other in oppositional trials due to the way in which responses are corrected. Correcting responses does two things to response-contingent data: It changes the scale of errors *and* it changes the distance between current stimulus and prior response. For stimulus-contingent data, only the scale of error is affected; the distance between current stimulus and prior stimulus does not change. This means that the stimulus and response biases will not perfectly mirror each other, even when both are corrected in the same way (see Supplementary Figure S1).

Although the negative bias value observed for a previous stimulus cannot be taken as evidence for a repulsive effect, we also cannot rule it out. If this is the case, then this repulsion must be weaker than the attractive effect observed from prior response, as the overall effect when stimulus and response are correlated (non-oppositional trials) is positive.

## Discussion

Using a task that experimentally separated the influence of previous stimuli and previous responses, we demonstrated that, for our stimuli, serial dependence was better quantified as an attraction to previous response than to the previous stimulus. This could be interpreted as supporting a decision-based account where observers tend to blend current and previous decisions without necessarily altering perception (Akaishi, Umeda, Nagase, & Sakai, 2014; Fritsche et al., 2017). Alternatively, response might well provide a better index of our perception than the stimulus itself, in which case an attraction between current and previous percepts may still account for serial dependence (Fischer & Whitney, 2014). The first study to use the response-contingent analysis implemented in the current experiment was motivated by the idea that response is more likely than objective stimulus values to resemble subjective perception (Fritsche, 2016). However, we would like to emphasize that our findings are agnostic toward the decision/perception debate.

One valid criticism of the current study might be that an apparent attraction toward the previous response could be an attraction to the final orientation of the previous response stimulus (i.e., the circles that appear when participants begin making their response). However, any orientation information expressed by these stimuli was not directly displayed, instead being expressed in the relative position of the dots, similar to adjustment stimuli used in previous studies (Ceylan et al., 2021; Cicchini et al., 2017). In addition, previous research has shown attraction to prior stimuli, not to prior response stimuli, when no response bar was presented (Manassi et al., 2017) or when participants were required to produce mirrored orientation responses as opposed to directly reproducing stimulus orientations (Cicchini et al., 2017).

The results of the current experiment provide direct experimental evidence that converges with the conclusions of earlier studies. Those previous studies used analysis techniques to disentangle the effects of prior stimulus and response. They suggest that it is response that drives attractive serial dependence (Moon & Kwon, 2022; Morimoto & Makioka, 2022; Pascucci et al., 2019; Sadil et al., 2024; Sheehan & Serences, 2023; Zhou et al., 2024). For example, Moon and Kwon (2022) reported attractive effects of prior responses and repulsive effects of stimuli. In this case, the stimulus was considered a proxy for sensory measurement and the response was considered a measure of participant perception.

Several studies have used observer error (the difference between stimulus and response) to explore the relative contribution of stimulus and response. By partitioning trials based on a median split of error in the previous trial, Sadil et al. (2024) investigated whether the current response was more similar to the prior response or the prior stimulus following inaccurate trials. The authors report a repulsive effect of prior stimulus and an attractive effect of prior response. Morimoto and Makioka (2022) reported similar results and suggested that the generally observed attractive effect of prior stimuli reflects the typically high correlation between stimulus and response, with the strong attractive effect of response overpowering any repulsion from stimuli.

Binary tasks with straightforward measures of correctness have also exploited errors. St. John-Saaltink et al. (2016) showed attraction to prior, incorrect responses as opposed to attraction to prior stimulus values. Similarly, Zhang and Alais (2020) reported comparable strength of assimilation to previous responses regardless of accuracy. The latter authors also examined the effect of previous stimulus and response on current responses and found an attractive effect of responses but no effect of prior stimuli. In both studies, attraction to incorrect responses was taken as a sign of attraction to perception of stimuli. In other words, response was seen as the better index of percept than stimulus.

Neural data has also supported the role of prior response in serial dependence. Ranieri, Benedetto, Ho, Burr, and Morrone (2022) used classification techniques to decode the previous response from current trial electroencephalogram data and found that higher decoding accuracy was correlated with the magnitude of serial dependence in participant responses. In contrast, decoding of the previous stimulus from current trial data was not possible. These results were taken to support the idea that the detectable trace of previous trial responses was driving serial dependence in the current trial. The authors suggested that responses might be expected to have more in common with the neural representation of stimuli than objective stimulus values. The motor aspect of response could also contribute to decoding accuracy, but the authors noted that this was minimized in their study by collecting verbal responses.

The current results contribute to the debate on how to think about serial dependence and suggest important factors to consider during analysis. In the experiments of Fischer and Whitney (2014) and in most subsequent research, the stimulus value displayed on the previous trial has been used to represent the point of attraction from the recent past. This assumes a straightforward relationship between prior stimulus and perception, which does make sense, as the displayed stimulus is a natural precursor for observer perception. However, we might not expect the stimulus value to always reflect perception perfectly, especially in cases where the orientation signal is degraded, such as lowered contrast (Cicchini, Mikellidou, & Burr, 2018; Fischer & Whitney, 2014; Pascucci et al., 2019) or increased noise (Gallagher & Benton, 2022). Stimuli that are more difficult to resolve should naturally lead to a greater disparity between stimulus and percept, which is consequently reflected in greater variation in response.

Assuming observers make responses that generally echo their perceptions, a participant's response value on the previous trial might well quantify previous perception more accurately than the previous stimulus. This is made evident by countless examples in the literature of participant perception differing from objective reality, with many studies aimed at inducing exactly this effect. One obvious example comes from motion adaptation, where perception of stimulus movement can be induced for static stimuli (Addams, 1834). In this case, taking non-moving stimuli to mean that observers *perceive* no motion would clearly be incorrect. Instead, taking the participant's response as a measure of their subjective state is the more reasoned approach (Dennett, 2003).

Additionally, in typical serial dependence experiments we might expect perception to differ from the displayed stimulus. Serial dependence tasks generally follow similar formats designed to elicit and measure this effect. Experimental trials do not usually exist in isolation (although see Manassi & Whitney, 2022) and the source of attraction on a *t* – 1 trial may itself be subject to serial dependence from the *t* – 2 trial. This would mean that observer perception on a *t* – 1 trial is likely to differ from presented stimulus values most of the time (assuming serial dependence to cause genuine differences in perception).

Response could be a straightforward reflection of perception, in which case the effect of the previous trial could in fact be an effect of previous perception. On the other hand, if some other factor intervenes between perception and response, such as memory failures or decision biases, then response may not perfectly capture observer perception.

More widely, debate in the literature has arisen around the idea of serial dependence as an attraction to previous decisions rather than an attraction to prior percept. If serial dependence is an attraction to prior decisions, then response-contingent analysis obviously makes more sense. If serial dependence is driven by the prior perception, then stimulus- or response-contingent analysis might be appropriate. Stimulus-contingent analysis may be permissible as a proxy for perception if we assume a tight coupling between stimulus and response (Zhang & Alais, 2020). However, as noted above, this is not necessarily true, especially in cases where stimuli incorporate noise (Gallagher & Benton, 2022) or low contrast (Cicchini et al., 2018; Fischer & Whitney, 2014; Pascucci et al., 2019).

Future experiments could explore whether the observed attraction to prior response varies with the level of stimulus noise. We might expect greater attraction to response with a lower signal-to-noise ratio. In this case, noise would be used to promote differences between the stimulus and response. Repeating the experiment with higher noise (perhaps not so high as to cause blind guessing) could demonstrate this. It might also be explored whether or not this varies with age, as a recent study has suggested (Zhou et al., 2024).

Regardless of whether an attraction to response reflects an attraction to percepts or decisions, response-contingent analysis may be the better way of analyzing serial dependence effects. Analyzing the influence of prior experience from a response-contingent perspective is a trivial adjustment to analyses that might generate clearer results in future studies of serial dependence. This can also circumvent issues that arise when stimuli elicit more varied responses, which can cause artifacts in analysis when stimulus values are used as the source of attraction (Gallagher & Benton, 2022). In conclusion, our results add to a growing body of evidence suggesting that serial dependence is best quantified as an attraction to previous responses.

## Supplementary Material

Supplement 1
