# Supplementary material for: Characterizing serial dependence as an attraction to prior response
Source: J Vis. 2024 Sep 26;24(9):16. doi: 10.1167/jov.24.9.16 (PMC11437684; doi:10.1167/jov.24.9.16)
Supplement: Supplement 1 [file jovi-24-9-16_s001.pdf]

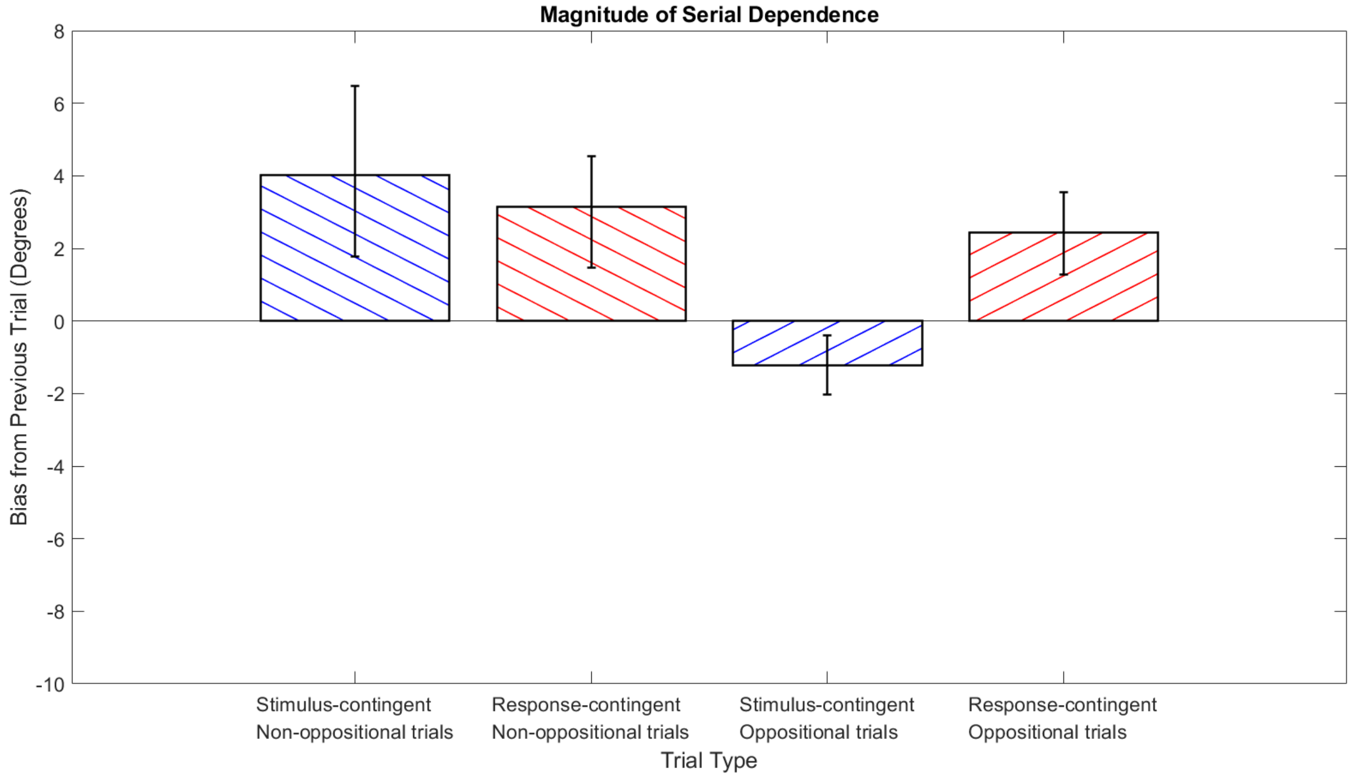

Supplemental Figure 1. Response- and stimulus-contingent biases for oppositional and non-oppositional trials. Error bars represent 95% confidence intervals. Response-contingent and stimulus-contingent forms of analysis corrected by residualisation.
